# Management of an infant with epidermolysis bullosa on invasive mechanical ventilation

**DOI:** 10.1590/1984-0462/2022/40/2020290

**Published:** 2021-07-05

**Authors:** Fabiola Fernandes, Luanda Bruno Pinheiro, Milena Siciliano Nascimento, Cristiane do Prado

**Affiliations:** a Hospital Israelita Albert Einstein, São Paulo, SP, Brazil.

**Keywords:** Respiration, artificial, Pediatrics, Epidermolysis bullosa, Physical therapy specialty, Ventilação mecânica, Pediatria, Epidermólise bolhosa, Fisioterapia

## Abstract

**Objective:**

To describe, for the first time in the pediatric population, the use of an effective technique to mobilize secretion in a patient whose disease imposes many care limitations.

**Case description:**

2-year-old infant with Epidermolysis Bullosa, dependent on mechanical ventilation after cardiorespiratory arrest. Infant evolved with atelectasis in the right upper lobe with restriction to all manual secretion mobilization techniques due to the risk of worsening skin lesions. We opted for tracheal aspiration in a closed system combined with expiratory pause, a technique only described in adult patients so far.

**Comments:**

This case report is the first to use this technique in a pediatric patient. The use of expiratory pause combined with tracheal aspiration not only optimized the mobilization of secretion, but it was also a safe tool for reversing atelectasis. Our case report brings an important result because it increases the possibilities of managing pediatric patients admitted to intensive care units, especially in situations of absolute contraindication for chest maneuvers.

## RESUMO

**Objetivo:**

Descrever pela primeira vez na população pediátrica a utilização de técnica eficaz para mobilização de secreção em paciente cuja patologia impõe muitas limitações no seu cuidado.

**Descrição do caso:**

Lactente de 2 anos e 4 meses com diagnóstico de epidermólise bolhosa dependente de ventilação mecânica pós-parada cardiorrespiratória evoluiu com atelectasia em lobo superior direito, com restrição a todas as técnicas manuais de mobilização de secreção em virtude do risco de agravamento das lesões de pele. Optou-se pela realização de aspiração traqueal em sistema fechado combinada à pausa expiratória, técnica até então só descrita em pacientes adultos.

**Comentários:**

O relato trata, portanto, do primeiro caso a utilizar essa técnica em paciente pediátrico. O uso da pausa expiratória combinada à aspiração traqueal não só otimizou a mobilização de secreção como também foi uma ferramenta segura para a reversão da atelectasia. O resultado revela-se importante, pois amplia as possibilidades do manejo de pacientes pediátricos internados nas unidades de terapia intensiva, principalmente em situações de contraindicação absoluta de manipulação torácica.

## INTRODUCTION

Epidermolysis bullosa (EB) is characterized by changes in intraepidermal or dermoepidermal adhesion that result in the appearance of blisters on the skin in response to minor trauma.^1^ Skin lesions make it difficult to manipulate the patient in daily care.^2^ Simple change in decubitus or passive joint mobilizations can be a challenge for the multidisciplinary team that assists these patients. In our case, the need for invasive mechanical ventilation and the presence of an artificial airway are added, which increases the risk of accumulation of secretion due to impairment of the mucociliary clearance mechanism.^3^

The respiratory system, from the nasal cavity to the respiratory bronchioles, is lined internally by a ciliated epithelium and submucosal glands and goblet cells, which are responsible for the production of respiratory mucus. The cilia are the propellants of mucociliary transport, and their coordinated beats direct the flow of mucus towards the hypopharynx, so the secretions are swallowed there.^4^ ^,^ ^5^ ^,^ ^6^ The ciliary beat is dependent on many interrelated factors, including the viscoelastic characteristics of mucus, humidity and temperature of inhaled air, respiratory volumes and flows.^6^ Patients on mechanical ventilation have most of these physiological factors altered, which favors the accumulation of secretion and can bring complications, such as increased airway pressures, carbon dioxide retention, changes in ventilation/perfusion ratio (V/Q), drop in arterial oxygen saturation (SpO_2_), ventilation-associated pneumonia, and atelectasis.^7^

Acting preventively and assisting the mechanism for mucociliary transport, the thoracic maneuvers that work to increase the expiratory flow and promote the direction of secretions to the most proximal airways are strongly addressed in the literature.^8^ ^,^ ^9^ More recently, techniques that use the mechanical ventilator as a tool for mobilization of secretion have been disseminated, with indications of its use in situations in which the patient cannot be manipulated a lot.^10^ ^,^ ^11^

The dynamics of the inspiratory and expiratory air flow generated by the ventilator configurations can contribute substantially to the movement of the mucus. Studies in animal and lung models have consistently shown that differences between inspiratory and expiratory flow can result in mucus migration, with possibility to eliminate or incorporate secretions. In order to move the cephalic mucus so that it can be easily removed by suction or cough, there must be a gain in the general expiratory flow.^12^

Both methods for open and closed endotracheal aspiration have advantages and disadvantages. In the first, patients are disconnected from the mechanical ventilator, which leads to hypoxemia and loss of lung volume. The second, however, removes less secretion. That being said, one of the main advantages of the closed aspiration method is that it avoids depressurization of the system, but maintaining the inspiratory flow impairs the removal of secretion.^13^ ^,^ ^14^ Thus, the use of expiratory pause associated with the closed aspiration system seems to be an interesting strategy to avoid depressurization of the system while guaranteeing secretion clearance.

In conditions such as EB and countless others that limit or prevent manipulation of the patient, tracheal aspiration with a closed system in association with expiratory pause emerges as an alternative with good applicability in the handling of patients in critical state admitted to the intensive care unit. This report aims to show this technique in children with EB.

## CASE REPORT

This report was submitted to the Research Ethics Committee, under CAAE number 34237120.8.0000.0071, and the Informed Consent Form (ICF) was signed by the legal guardian of the patient. Clinical research was carried out in accordance with the Declaration of Helsinki.

Female patient, 2 years and 4 months old, with Recessive Dystrophic Epidermolysis Bullosa Syndrome. Halogenous bone marrow transplant had been performed in June 2019, with bone marrow grafting or marrow “handle” in December 2019.

Diagnosed with post-transplant autoimmune hemolytic anemia (Hb: 4.4g/dL and Ht: 8.2%) in January 2020, requiring hospitalization for pulse therapy. She evolved negatively, requiring orotracheal intubation (OTI), followed by two cardiorespiratory arrests (CRA). Patient was seen at the institution without sedation to assess the evolution of the neurological condition after CRA, showing no response to pain and absence of cough reflex. She was hemodynamically stable, without vasoactive drugs, OTI under mechanical ventilation in assisted/controlled mode and controlled pressure (AC/CP): controlled pressure (CP): 14; end expiratory pressure (PEEP): 8; respiratory rate (RR): 30; FIO_2_: 50%; inspiratory time (Tinsp): 0.75 seconds; and tidal volume (TV): 11mL/kg.

**Figure 1:**
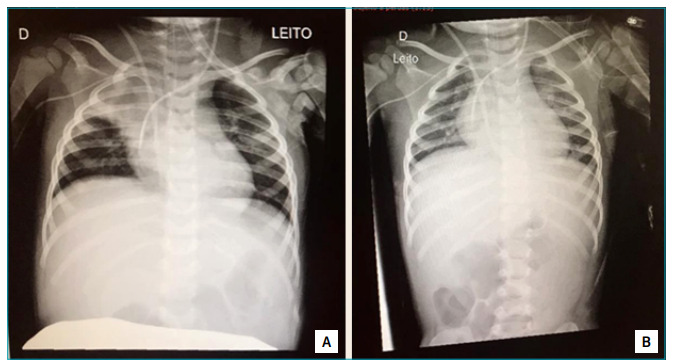
Chest radiography of patient with epidermolysis bullosa. (A) Hypotransparency at the right apex; (B) Improvement of hypotransparency after expiratory pause maneuvers during closed system aspiration.

Chest X-ray was suggestive of atelectasis in the right upper lobe (Figure 1A). There were clinical restrictions for conventional bronchial hygiene maneuvers and the patient maintained the absence of cough reflex even during aspirations.

It was established that during respiratory physical therapy sessions, expiratory pause maneuvers would be performed on the mechanical ventilator to optimize the removal of secretions, since any type of bronchial hygiene maneuver was contraindicated due to skin lesions.

The aspirations were performed with a closed system, being the first aspiration of the service performed without the expiratory pause and the following aspirations associated with expiratory pause for five seconds in the mechanical ventilator. The secretion during aspiration without expiratory pause was minimal or none, and with the expiratory pause, it was voluminous and semi-thick.

A control X-ray, after three physical therapy sessions with expiratory pause, showed total reversal of atelectasis in the right apex (Figure 1B).

## DISCUSSION

This is the first case report of a pediatric patient being submitted to tracheal aspiration with a closed system combined with expiratory pause, an effective technique for mobilizing secretion in patients whose pathology imposes many care limitations.

The closed suction system has the ability to reduce the risk of pulmonary infections because there is no direct exposure of the airways or direct handling of the suction tube by a health professional, in addition to reducing episodes of desaturation and alveolar collapse.^14^ ^,^ ^15^ ^,^ ^16^ However, it does not seem to be as effective for secretion removal as the open system.

The presence of an artificial airway, the effect of paralyzing agents, ventilation with high concentrations of oxygen, lesions of the tracheobronchial mucosa induced by tracheal aspiration and inadequate humidification seem to be the main determinants of changes in mucociliary function in mechanically ventilated patients.^17^ ^,^ ^18^ With an excessive production of mucus, these factors increase the risk of secretion retention, pulmonary infection and the development of atelectasis due to obstruction.^17^

Mucus transport can be influenced by variations in inspiratory and expiratory flows,^19^ ^,^ ^20^ which are the basis for the bronchial hygiene techniques performed by the physical therapist in patients with hypersecretion. Manual techniques, widely used in the pediatric population, such as vibration and change in the expiratory flow, also alter the pleural pressure, favoring the movement of mucus.^21^ In the case reported, the manual techniques used to mobilize the mucus had absolute contraindications because of the patient’s underlying disease, which is characterized by the development of blisters on the skin as a result of minimal friction.

In addition to manual techniques, some authors have suggested a mechanical ventilator as a tool to increase inspiratory flow, such as adjusted hyperinflation. This technique aims to increase alveolar ventilation and facilitate the mechanism of cough, aiding in the transport of mucus.^22^ ^,^ ^23^ Martins et al., in a study with 31 adult patients, observed that closed system endotracheal aspiration combined with an expiratory pause increased the amount of secretion aspirated, leading to the hypothesis that an expiratory pause would stabilize air pressure. It would result in greater effectiveness of negative pressure during tracheal aspiration and, thus, be a possible alternative to stabilize airway pressure during the procedure, increasing efficiency and allowing uninterrupted ventilation.^24^

In the reported case, some factors favored a greater accumulation of secretion in this patient, besides the difficulty in handling these secretions, which led to the appearance of atelectasis. The presence of lobar atelectasis compromises gas exchange, one of the main risk factors for extubation failure in the pediatric population.^12^ Thus, the use of the expiratory pause tool combined with tracheal aspiration helped reverse the atelectasis and, consequently, decrease the time of mechanical ventilation.

Respiratory complications are common in patients admitted to intensive care units, especially those who require orotracheal intubation and mechanical ventilation. This report opens the possibility that expiratory pause combined with tracheal aspiration can be safely used in the pediatric population to optimize the mobilization of secretion with greater effectiveness in pulmonary hygiene. The performance of a prospective study to confirm these findings is important.
